# Modular prediction of protein structural classes from sequences of twilight-zone identity with predicting sequences

**DOI:** 10.1186/1471-2105-10-414

**Published:** 2009-12-13

**Authors:** Marcin J Mizianty, Lukasz Kurgan

**Affiliations:** 1 Department of Electrical and Computer Engineering, University of Alberta, Edmonton, Canada

## Abstract

**Background:**

Knowledge of structural class is used by numerous methods for identification of structural/functional characteristics of proteins and could be used for the detection of remote homologues, particularly for chains that share twilight-zone similarity. In contrast to existing sequence-based structural class predictors, which target four major classes and which are designed for high identity sequences, we predict seven classes from sequences that share twilight-zone identity with the training sequences.

**Results:**

The proposed MODular Approach to Structural class prediction (MODAS) method is unique as it allows for selection of any subset of the classes. MODAS is also the first to utilize a novel, custom-built feature-based sequence representation that combines evolutionary profiles and predicted secondary structure. The features quantify information relevant to the definition of the classes including conservation of residues and arrangement and number of helix/strand segments. Our comprehensive design considers 8 feature selection methods and 4 classifiers to develop Support Vector Machine-based classifiers that are tailored for each of the seven classes. Tests on 5 twilight-zone and 1 high-similarity benchmark datasets and comparison with over two dozens of modern competing predictors show that MODAS provides the best overall accuracy that ranges between 80% and 96.7% (83.5% for the twilight-zone datasets), depending on the dataset. This translates into 19% and 8% error rate reduction when compared against the best performing competing method on two largest datasets. The proposed predictor provides accurate predictions at 58% accuracy for membrane proteins class, which is not considered by majority of existing methods, in spite that this class accounts for only 2% of the data. Our predictive model is analyzed to demonstrate how and why the input features are associated with the corresponding classes.

**Conclusions:**

The improved predictions stem from the novel features that express collocation of the secondary structure segments in the protein sequence and that combine evolutionary and secondary structure information. Our work demonstrates that conservation and arrangement of the secondary structure segments predicted along the protein chain can successfully predict structural classes which are defined based on the spatial arrangement of the secondary structures. A web server is available at http://biomine.ece.ualberta.ca/MODAS/.

## Background

Protein function, regulation, and interactions can be learned from their structure [1,2], which motivates development of novel methods for the prediction of the protein structure. These predictions concern various levels and aspects of the protein structure including the tertiary structure [3,4], solvent accessibility, depth, flexibility and packing of residues [5], and secondary structure [6]. In contrast to the tertiary structure that describes position of each of the protein's atoms, the secondary structure simplifies the protein structure to a set of spatially local folding patterns that include α-helices, β-strands and coils. The spatial distribution of these local patterns determines the overall, three-dimensional shape of proteins in which individual secondary structures interact with each other creating more complex structures such as parallel or antiparallel β-sheets, β-barrels, and others. In spite that final product is complex, protein structures can be categorized into a few structural classes depending on the amount, types and spatial distribution of the secondary structures found in their fold.

Knowledge of the structural class is shown to stimulate the development of methods for identification of other structural and functional characteristics of proteins [7]. Examples include prediction of protein unfolding rates [8], characterization and prediction of folding rates [9-11], quantification of the relation between chain lengths and folding rates of two-state proteins [12], prediction of DNA-binding sites [13], discrimination of outer membrane proteins [14], fold prediction [15], secondary structure and secondary structure content prediction [16,17], reduction of the conformation search space [18] and implementation of a heuristic approach to find tertiary structure [19], to name just a few. At the same time, the structural classes are known for a relatively small number of proteins. The most recent release 1.75 of SCOP database [20,21] includes 110,800 protein domains with the annotated classes, while release 36 of the NCBI's RefSeq database [22] includes 8,181,910 non-redundant protein sequences. The main reason for this wide gap is unavailability of protein structure, which is used to assign the structural class, for the significant majority of the known protein sequences. To this end, an accurate and automated method for classification of sequences into the corresponding structural classes would provide assistance when the structural class in unknown for a given chain.

Template-based modeling, which is successfully used to predict the tertiary structure, is based on an assumption that similar sequences (usually defined as sequences with similarity of above 30%) share similar structures [23-25]. Prediction methods that rely on the sequence alignment [26,27] usually perform relatively poorly when sequences with high identity are not available. More specifically, over 95% of protein chains characterized by low, 20-25%, pairwise identity, which is referred to as the twilight-zone similarity, have different structures [28], which substantially reduces accuracy of the corresponding predictions. We observe that about 40% of sequences for which the tertiary structure was deposited to Protein Data Bank (PDB) [29] in 2005 share twilight-zone pairwise similarity with any sequence deposited in the PDB before 2005 [30], which motivates development of the prediction methods for these challenging chains. Further motivation comes from the fact that finding similar folding patterns among the proteins characterized by low sequence identity is beneficial for the reconstruction of the tertiary structure [31,32]. Researchers have observed that pairs of sequences with low identity may share similar folding patterns or overall structure [33-35] and they can be used to predict tertiary structure [3,36,37]. The accurate alignment of the distant homologues (proteins with similar structures and sequences that share low identity) is still a challenging problem in spite of many years of research in this area [36,37]. We note that structurally similar proteins that share low sequence identity can be found based on coarse grained classifications such as the structural classes that are addressed in this work. We believe that the proposed method could find applications in the detection of remote homologues.

### Protein structural class

**Figure 1:**
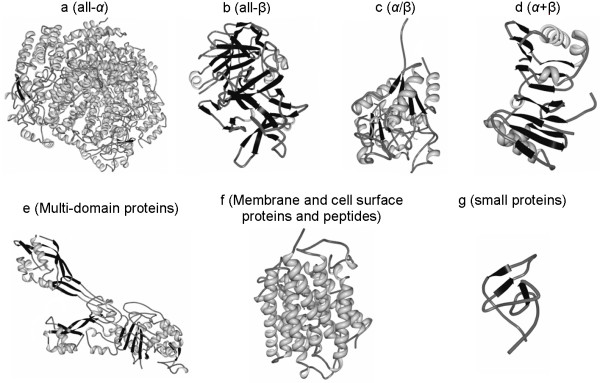
**Cartoon structures of proteins that cover the seven structural classes defined in the SCOP database**. Panel a shows structure of protein with PDB identifier 1mty, b for 1a8d, c for 2f62, d for 2bf5, e for 1vqq, f for 1u7g, and g for 4hir. Helices are shown in light grey, coils in dark gray, and strands in black.

Two databases which classify protein structures include SCOP (**S**tructural **C**lassification **o**f **P**roteins) [20,21] and CATH (**C**lass, **A**rchitecture, **T**opology and **H**omologous superfamily) [38,39]. The former database relies on a manual process to classify the structures while the latter applies a combination of automated and manual procedures. The first level of the classification hierarchy in both databases is the structural class. The SCOP distinguishes seven classes where the four major classes, which cover almost 90% of all SCOP entries, are all-α, all-β, α+β and α/β. The two former classes include structures dominated by α-helices and β-strands, respectively. The two latter classes correspond to structures that include both helices and strands where in the case of the α+β class these secondary structures are segregated, whereas for α/β class the structures are interspersed. The three remaining classes include multi-domain proteins, membrane and cell surface proteins and peptides, and small proteins. The multi-domain proteins consist of several domains where each domain may belong to a different class while the small proteins have short sequences and their secondary structures do not fit the definition of the other classes. We note that in spite of the fact that membrane proteins are relatively common their coverage in SCOP database is relatively low as it is difficult to obtain their structure [40]. The SCOP also includes four supplementary categories, i.e., coiled coil, designed, and low resolution, proteins; and peptides, but they have limited practical implications. Figure 1 shows representative structures for the seven classes in the SCOP database.

CATH database defines only four classes that include mainly α, mainly β, mixed α-β, and proteins with few secondary structures. In this work we address the SCOP based classification as it further subdivides the mixed proteins, defines several important additional classes such as membrane and multi domain proteins, and since most of the existing structural class prediction methods are also based on this definition of the structural classes. Moreover, the structural classes defined in CATCH are relatively easy to predict based on the secondary structure content of a protein, which in turn could be predicted using existing content prediction methods [41,42]. This is in contrast to the classification in the SCOP database where more complex information, such as relative amount and spatial position of the secondary structures, is used to assign classes [43].

### Related work

The manual assignment of structural classes performed in SCOP is based on spatial arrangement of secondary structure segments which is inspected using the tertiary structure. We aim at building an automated method which makes the class predictions based solely on the protein sequence. Prediction is typically performed in two steps: 1) the variable-length sequences are converted into a fixed-length feature vectors; 2) the feature vectors are inputted into a classification algorithm to generate the class prediction.

Due to a relatively large existing body of research in this area the following review concentrates on recent methods. The reader is referred to a review by Chou [7] that provides further details on older methods and that motivates the development of the structural class prediction methods.

Majority of the developed methods use relatively simple features such as composition vector, pseudo amino acid (AA) composition [44], composition of short polypeptides, sequence itself and other features obtained from AA sequence [45-71]. Several recent methods use more advanced feature vectors [30,72-78] which are based on the AA sequence and/or PSSM profile computed using PSI Blast [26]. A recently explored alternative is to construct features based on the predicted secondary structure. This approach was used in SCPred algorithm [79], which up to date provides favorable prediction quality on datasets characterized by the twilight-zone similarity.

A wide range of classification algorithms was used to perform the predictions. They include component coupling [70], neural network [80], Bayesian classifier [81], logistic regression [30,58,72,73], decision tree [46,54], covariant or linear discriminant algorithm [57,64,65,77,78], principal component analysis [55], nearest neighbor [52,67-69], rough sets [49] and support vector machine (SVM) [45,47,48,50,53,54,61,62,66,72,74,75,79,82]. Recent works also explored more complex classification models such as ensembles [72], bagging [54,63], and boosting [56,59,71]. Overall, we observe that SVM is the most popular and the best-performing classifier for this task [79].

The prediction quality of these methods varies widely depending on the datasets [73]. The methods which were tested on datasets with relatively high sequence identity report accuracies of close to or over 90% [45,47-50,53-55,57,58,61-66,68-72,74-77]. The tests on the dataset characterized by the low, twilight-zone identity show accuracies between 50 and 70% [30,45,52,56,59-61,67,72,73,75,78,79,81] with only one approach, namely SCPred, that obtains accuracies 80% [79]. We concentrate on the latter problems as they are more challenging and have implications in the context of the remote homology detection.

The above methods considered only the four major classes from the SCOP database, which was motivated by a relatively small number of proteins in the remaining classes. At the same time, recent years observed a substantial increase in the size of the SCOP database which doubled in size between 2003 and 2007, and which currently includes over 100,000 protein domains. Even when considering a small subset of the protein domains in SCOP which is characterized by the twilight-zone similarity, we note that the current SCOP includes sufficient number of proteins for the smaller three classes to allow for the development of a prediction system.

There are only two methods that addressed prediction of the seven classes [83,84]. The first method predicts the four main classes and multiple domain, small protein, and peptide classes [84]. This differs from prediction targets of MODAS which additionally considers membrane and cell surface proteins as a part of the peptide class. This method is shown to achieve accuracy of over 90% for a low-identity dataset by using a large library of reference functional sequence motifs from the InterPro database [85]. This resulted in the feature vector with 7,785 features where each feature denotes occurrence of a given motif in the input sequence. Although this method is characterized by good prediction quality, we note that it does not provide a web server, is difficult to implement due to the excessive number of used features, and was not redesigned in spite of the updates in the InterPro database (the current release 19 of InterPro includes 17,412 motifs while the authors used version 6.2 from April 2003). We also note that the usage of such a large number of features results in an ill-defined problem in which the number of classification instances (protein chains) is smaller than the number of features. The second, more recent method [83] uses a complex representation of the protein sequence that includes pseudo AA composition, evolutionary conservation information, and physicochemical properties of AAs, and the SVM classifier to perform predictions. It achieves accuracy of 57.4% for a dataset with the twilight-zone identity. We perform an empirical side-by-side comparison with this method.

Although structural class predictors usually do not consider membrane and multi domain classes, such predictions could be addressed using methods designed specifically for these classes. We refer the reader to recent review articles concerning methods that are available for the prediction of membrane proteins [86-88] and for the domain prediction [89,90]. These developments are motivated by the availability of specialized databases for the membrane [91] and multi-domain proteins [92]. The abovementioned methods could discriminate chains in the corresponding class from all other chains, and they could be used to either pre-filter the chains or post-process results of the proposed MODAS method. More specifically, once a given chain is known to be a membrane protein, specialized predictors could be used to further categorize its membrane proteins type [14,24]. Similarly, the predicted multi-domain proteins could be processed by the available methods to predict the domain boundaries [90].

### Motivation and goals

All but two existing structural class predictors consider only the four major classes, while the remaining three classes are also important and their prediction should be addressed. For instance, while approximately 20 to 35% of the proteins encoded by an organism's genome are membrane proteins [93], they are not covered in the four major classes. As mentioned above, the main reason for their under-representation in the SCOP database is that they are difficult to crystallize and as a result only a small number of membrane protein structures are known [40]. We also note that the current methods are relatively weak in the context of the sequence representation. Most of the methods compute the representation directly from the sequence, only a handful of them use sequence-derived information such as multiple alignment [75,79,83] and predicted secondary structure [79], and there were no attempts to combine residue conservation computed from the alignment and the secondary structure. At the same time, the usage of the predicted secondary structure results in improved prediction quality for the low identity datasets [79], and numerous prior studies have demonstrated that evolutionary information generated with PSI Blast [26] is more informative than the sequence itself [94-96]. Moreover, most of the existing predictors achieve good quality for datasets with high sequence similarity, while results on the datasets with the twilight-zone pairwise similarity are generally characterized by a relatively low, <70%, accuracy (with the exception of one method that obtains close to 80% accuracy). At the same time, a solution that accommodates for the low sequence identity could have important applications for the tertiary structure prediction [3,35,36]. Finally, the existing methods are fixed to a given set of classes, while a modular design would allow the user to choose how many and which classes should be considered for the prediction. The latter is a particularly attractive feature for a method that would address all 7 classes, i.e., the user could choose which subset of classes, including the four major classes, should be considered for a given prediction. We also observe that current methods use the same feature-based sequence representation for prediction of all classes. In the modular design a separate predictor is created for each class and the results of these predictors are combined together. This allows for the design of a specialized sequence representation for each class.

Our goal is to develop a novel, modular method that predicts the seven structural classes from the protein sequences. The proposed modular approach to structural class prediction (MODAS) exploits sequence and sequence-derived information to generate input for the classifier. More specifically, MODAS is the first to combine both the multiple sequence alignment profiles and the predicted secondary structure to generate features that are fed into a set of seven SVM classifiers. Our design concentrates on datasets that include sequences characterized by low, twilight-zone similarity and we aim at providing prediction quality that is competitive or better than the quality offered by the existing methods.

## Methods

### Datasets

We use total of 7 datasets to design and test the proposed method. We utilized version 1.73 of the ASTRAL database [97], which is a subset of the sequences from the SCOP database characterized by a certain similarity threshold, to derive two datasets. We selected the ASTRAL database with < 20% sequence similarity that includes 6264 sequences where 1280 of them belong to the all-α class, 1324 to all-β, 1495 to α+β 1527 to α/β, 106 to multi-domain proteins, 138 to membrane and cell surface proteins and peptides, and 394 to small proteins class. We randomly divided this set into two equal size subsets, one that was used as the training set (ASTRAL_training_) and the second that was used as the test set (ASTRAL_test_). The ASTRAL_training _set was used to design the proposed method, which includes features and classifier selection and parameterization of the classifiers. The ASTRAL_test _set was used to perform an independent (from the training set) validation of the proposed method. Both of these datasets are available at http://biomine.ece.ualberta.ca/MODAS/.

We also selected 4 widely used low sequence identity benchmark datasets to provide a comprehensive and unbiased comparison with the existing prediction methods. The D2230 dataset includes 2230 sequences extracted using ASTRAL version 1.63 using 20% identity threshold which was used to test the most recent method for prediction of the 7 classes [83]. We use this dataset to perform a side-by-side comparison with the method by Chen and colleagues [83]. The remaining 3 datasets are used to compare against methods that address prediction of the four major structural classes. The 25PDB dataset, which includes 1673 sequences which share twilight-zone pairwise similarity, was taken from [73] and two datasets D1189 and D675 were taken from [81] and [75] and include 1189 sequences with up to 40% pairwise identity and 675 sequences with up to 30% pairwise identity, respectively. The latter three datasets are the most commonly used benchmark sets that include low identity sequences and they allow for a side-by-side comparison with a wide selection of recent methods for the prediction of the four major structural classes.

**Figure 2:**
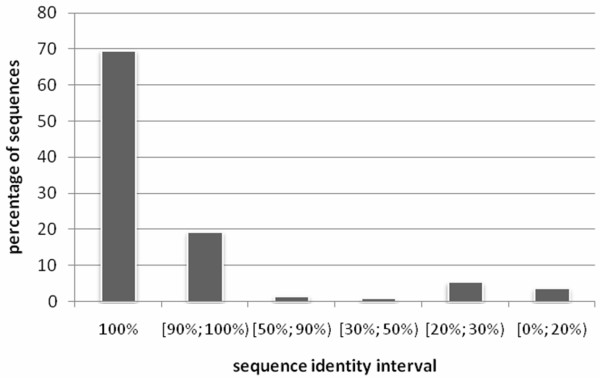
**Distribution of sequences with respect to their maximal pairwise sequence identity in the D498 dataset**.

Finally, we include one larger benchmark dataset, namely D498, which have been proposed in [70] and which includes a set of sequences that were not filtered with respect to their similarity. We include this dataset to demonstrate the quality of the proposed method when compared with a wider range of predictors which were tested on datasets with unspecified sequence identity. We explore the distribution of the sequence identity in this dataset to compare it with the other 6 datasets. For each chain we compute maximal sequence identity with all remaining sequences in the dataset. We chose the maximal values since the empirical tests are based on the jackknife strategy in which all but one sequence are used to predict the class for the remaining chain. We generate pairwise sequence alignments using Smith-Waterman algorithm [98] with Gotoh's improvement [99] and for each sequence we report the highest obtained score. The number of matching residues in the alignment is divided by the length of the query sequence including the gaps/insertions; a result of 100% sequence identity means that there were no gaps/insertions and that a query sequence was a substring of one of the sequences in the dataset. Figure 2 shows the distribution of sequences in the D498 dataset based on the sequence identity. Almost 70% of sequences from this dataset have 100% sequence identity and around 89% have identity of above 90%. This means that using the jackknife test, 89% of the tested sequences have are at least one very similar sequence in the training part of the dataset. This explains higher predictive performance on this dataset when compared with results on the remaining datasets with controlled, low sequence identity (see Results and Discussion section).

### Overall design

**Figure 3:**
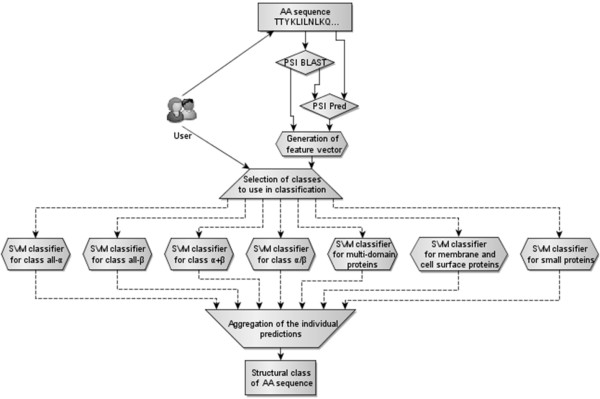
**Diagram of the proposed MODAS method**.

The input protein sequence is first processed by PSI Blast to obtain the position specific scoring matrix (PSSM) and by PSI Pred [100] to predict secondary structure. We selected PSI Pred due to its successful application in the SCPred method [79] and since this predictor enjoys a widespread use in prediction of a variety of related structural properties of proteins including template-based tertiary structure prediction [37], and prediction of beta-turns [101], residue depth [102], protein fold [31], and contact orders [103], to name just a few. Next, the sequence, the PSSM and the predicted secondary structure are converted into a set of features that are fed into seven classifiers (user can opt to use a subset of the classifiers), where each classifier corresponds to one of the seven SCOP classes. We performed feature selection to find a suitable set of features for each structural class. We also considered several different classifier types and selected the one that provides the best prediction quality for a given class. The seven classifiers generate a probability of classification into the corresponding class and these probabilities are aggregated to predict the final outcome. The aggregation is based on a simple max operator, i.e., we predict the class that corresponds to the highest probability. Although more complex aggregations could be conceived, this approach is motivated by the necessity to assure modularity of the predictor, i.e., the aggregation should work for every subset of the considered seven classes. The overall design of the proposed MODAS method is shown in Figure 3.

The design of the proposed method concerns development and selection of the features which best describe each of the classes and a classifier which provides the best predictive performance. The feature and classifier selection is based on 10-fold cross validation on the ASTRAL_training _dataset to assure that the design is independent of the other datasets and, at the same time, that it generalizes into the other datasets. The methods were written in JAVA language and we utilized the WEKA workbench [104] in this research.

### Feature vector

The three sources of data used to generate the features include the protein sequence, the PSSM matrix generated with PSI Blast and the secondary structure predicted from the sequence using PSI Pred. The PSSM matrices were built using the nr (non-redundant) dataset [105], as of October 2008. The quality of the matrix, and consequently the quality of the proposed method, depends on the size of the dataset used. Prior results demonstrate that larger number of diverse sequences in the database leads to more accurate evolutionary information, which in turn was shown to improve secondary structure predictions [106]. This suggests that subsequent retraining of the MODAS method at a later time using updated, larger nr database may potentially lead to better predictive performance. Besides features that were based on counting individual AAs, the AAs were grouped according to their physicochemical properties including polarity (R group), hydrophobicity, structure-preserving mutations (exchange groups), and their ability to be electron donors or acceptors, see Table 1. We also used these groupings in connection with the predicted secondary structure, i.e., amino acids were grouped based on their secondary structure and a given property. Finally, we considered combining information coming from the predicted secondary structure with the multiple alignments.

**Table 1:** The property groups used to aggregate similar amino acids.

R groups		Electronic groups	
Non-polar aliphatic	A, I, L, V	Donors	A, D, E, P

Glycine	G	Weak donors	I, L, V

Non-polar	F, M, P, W	Acceptors	K, N, R

Polar uncharged	C, N, Q, S, T, Y	Weak acceptors	F, M, Q, T, Y

Polar charged	D, E, H, K, R	Neutral	C, G, H, S, W

**Hydrophobicity groups**		**Exchange groups**	

Hydrophobic	A, C, F, I, L, M, P, V, W, Y	Group 1	H, R, K,
		
		Group 2	D, E, N, Q,
		
		Group 3	C

Hydrophilic	D, E, G, H, K, N, Q, R, S, T	Group 4	S, T, P, A, G
		
		Group 5	M, I, L, V,
		
		Group 6	F, Y, W

The features are divided into five sets: 1) features generated directly from the sequence; 2) features computed from the PSSM matrix; 3) features generated by combining information from PSSM and the predicted secondary structure; 4) features obtained from the predicted secondary structure, which are based on the features utilized in the SCPred method [79]; and 5) novel features based on the predicted secondary structure which describe collocation of helical and strand segments.

#### Features based on the AA sequence (39 features)

These features describe basic characteristics of the input sequence, such as length, AAs composition and composition of property groups. They include:

- *SeqLen *- the length of a sequence. (1 feature)

- *Comp_AA*_*i *_= , the number of AA_*i *_in the sequence (also called composition of AA_*i*_) normalized by the sequence length where *i *= 1, 2,..., 20 and AA_*i *_stands for *i*^th ^AA type. (20 features)

- *Comp_GR_GR*_*jk *_= , the number of AAs in the sequence belonging to *GR*_*jk *_where *j *∈ {R group, Electronic group, Hydrophobicity group, Exchange group} and *k *is a particular subgroup (e.g., hydrophobic and hydrophilic), see Table 1, normalized by the sequence length. (18 features)

#### Features based on the PSSM matrix (196 features)

The PSI Blast provides two position specific scoring matrices; one contains conservation scores of a given AA at a given position in a sequence, denoted as PSSMcons_*lm*_, and the other provides probability of occurrence of a given AA at given position in the sequence, denoted as PSSMprob_*lm *_where *l *= 1, 2...SeqLen denotes the position in the sequence and *m *= 1, 2,..., 20 denotes one of the substitution positions that correspond to the twenty AAs (columns in the PSSM matrix). We normalized the conservation scores (PSSMcons_*lm *_values) using max-min normalization where min and max equal -8 and 13, respectively. The PSSMprob_*lm *_values are already normalized by the PSI Blast. The matrix values were aggregated either horizontally (along *m*) or vertically (along *l*) to obtain a fixed length feature vector. This feature set, which quantifies evolutionary information of individual AA types and grouping of AAs according to the property groups, includes the following features:

- *Ach_CS_{AA*_*i*_} = , sum of all normalized PSSMcons_*lm *_values ("Ach_CS" stands for achieved conservation scores), where *l *includes only positions of AA_*i *_(along the sequence only the positions of AA_*i *_residues where considered) and *m *= AA_*i *_(column that corresponds to AA_*i*_), divided by the sequence length. (20 features)

- *Max_CS_{AA*_*i*_} = , sum of maximal, over *m*, PSSMcons_*lm *_values, where *l *includes only positions of AA_*i*_, divided by the sequence length. (20 features)

- *Max-Ach_CS_{AA*_*i*_} = , sum of differences between maximal PSSMcons_*lm *_(over *m *values) and PSSMcons_*li *_values where *l *includes only positions of AA_*i*_, and *i *= AA_*i *_(the difference between the maximal and the achieved values), divided by the sequence length. (20 features)

- *Ach_Prob_{AA*_*i*_} = , sum of all normalized PSSMprob_*lm *_values ("Ach_Prob" stands for achieved probability of occurrence), where *l *includes only positions of AA_*i *_and *m *= AA_*i*_, divided by the sequence length. (20 features)

- *Max_Prob_{AA*_*i*_} = , sum of maximal, over *m*, PSSMprob_*lm *_values, where *l *includes only positions of AA_*i*_, divided by the sequence length. (20 features)

- *Max-Ach_Prob_{AA*_*i*_} = , sum of differences between maximal PSSMprob_*lm *_(over *m *values) and PSSMprob_*li *_values where *l *includes only positions of AA_*i*_, and *i *= AA_*i *_(the difference between the maximal and the achieved values), divided by the sequence length. (20 features)

- *CSSeq_{AA*_*i*_} = , sum of normalized PSSMcons_*lm *_values where *l *= 1, 2...SeqLen and *m *= AA_*i*_, divided by the sequence length (average conservation score of AA_*i*_, for the whole sequence). (20 features)

- *CSSeq_GR_{GR*_*jk*_} = , sum of normalized PSSMcons_*lm *_values where *l *= 1, 2...SeqLen and *m *= *GR*_*jk *_(all AA types that belong to *GR*_*jk*_) divided by the sequence length. (18 features)

- *Ent_{AA*_*i*_} = , entropy of PSSMprob_*lm *_values, for *l *= 1, 2...SeqLen and *m *= AA_*i*_. (20 features)

- *Avg_Prob_GR_{ GR*_*jk*_} = , average PSSMprob_*lm *_values where *l *= 1, 2...SeqLen and *m *= *GR*_*jk *_(all AA types that belong to *GR*_*jk*_) divided by the sequence length. (18 features)

The *Ach_CS_{AA*_*i*_*}*, *Max_CS_{AA*_*i*_*}*, *Max-Ach_CS_{AA*_*i*_*}*, *Ach_Prob_{AA*_*i*_*}*, *Max_Prob_{AA*_*i*_*}*, and *Max-Ach_Prob_{AA*_*i*_*} *features aggregate information along the sequence by the AA type. The *CSSeq_{AA*_*i*_*}, CSSeq_GR_{GR*_*jk*_*}, Ent_{AA*_*i*_}, and *Avg_Prob_GR_{GR*_*i*,*j*_*} *aggregate the values along the columns of the PSSM.

#### Features based on the PSSM matrix in combination with the predicted secondary structure (486 features)

The third feature set is analogous to the features based on the PSSM matrix, but instead of aggregating the values by AA type, they are aggregated either by the type of the secondary structure predicted with PSI Pred or by the combination of the AA type and the predicted secondary structure. These features quantify conservation of predicted secondary structures, as well as the conservation for individual AA types and grouping of AAs according to the property groups that are in a given predicted secondary structure. This feature set consists of:

- *Ach_CS_{AA*_*i*_*}*, *Max_CS_{AA*_*i*_*}*, *Max-Ach_CS_{AA*_*i*_*}*, *Ach_Prob_{AA*_*i*_*}*, *Max_Prob_{AA*_*i*_*}*, and *Max-Ach_Prob_{AA*_*i*_*} *are redefined as *Ach_CS_{SS*_*n*_*}*, *Max_CS_{SS*_*n*_*}*, *Max-Ach_CS_{SS*_*n*_*}*, *Ach_Prob_{SS*_*n*_*}*, *Max_Prob_{SS*_*n*_*}*, and *Max-Ach_Prob_{SS*_*n*_*}*, respectively, where instead of using 20 *AA*_*i *_we aggregate by the predicted three state secondary structure *SS*_*n *_= *{H, E, C}*. (6*3 = 18 features)

- *Ach_CS_{AA*_*i*_*}*, *Max_CS_{AA*_*i*_*}*, *Max-Ach_CS_{AA*_*i*_*}*, *Ach_Prob_{AA*_*i*_*}*, *Max_Prob_{AA*_*i*_*}*, and *Max-Ach_Prob_{AA*_*i*_*} *are redefined as *Ach_CS_{SS*_*n*_*}_{AA*_*j*_*}*, *Max_CS_{SS*_*n*_*}_{AA*_*j*_*}*, *Max-Ach_CS_{SS*_*n*_*}_{AA*_*j*_*}*, *Ach_Prob_{SS*_*n*_*}_{AA*_*j*_*}*, *Max_Prob_{SS*_*n*_*}_{AA*_*j*_*}*, and *Max-Ach_Prob_{SS*_*n*_*}_{AA*_*j*_*}*, respectively, where we aggregate PSSMcons_*lm*_/PSSMprob_*lm *_values by *l *that corresponds to positions of AA_*i *_that are predicted as *SS*_*n*_. (6*3*20 = 360 features)

- *CSSeq_GR_{GR*_*jk*_*} *and *Avg_Prob_GR_{GR*_*jk*_*} *are redefined as *CSSeq_GR_{GR*_*jk*_*}_SS_{SS*_*n*_*} *and *Avg_Prob_GR_{GR*_*jk*_*}_SS_{SS*_*n*_*}*, respectively, where we aggregate PSSMcons_*lm*_/PSSMprob_*lm *_values by *l *that corresponds to a given *SS*_*n*_. (2*3*18 = 108 features)

#### Features based on the predicted secondary structure (144 features)

The fourth feature set, which was computed based on the output of PSI Pred, describes the content of the predicted secondary structures and distribution of the predicted secondary structures segments aggregated based on segments length and by grouping of AAs according to the property groups. This set consists of:

- *Content_{SS*_*n*_} = , the number of residues predicted as *SS*_*n *_where *l *= 1, 2...SeqLen, divided by the sequence length. (3 features)

- Content_*{SS*_*n*_*}_GR_{GR*_*jk*_} = , the number of residues predicted as *SS*_*n *_and that belong to *GR*_*jk *_where *l *= 1, 2...SeqLen, divided by the sequence length. (3*18 = 54 features)

- *SegCount_{E,H}_L{L*_*i*_} = , the number of helix or strand segments which contain at least *L*_*i *_= 2, 3, .. 20 AAs divided by the total number of helix and strand segments in the input protein chain. (2*19 = 38 features)

- *SegCount_C_L{L*_*i*_} = , the number of coils which contain at least *L*_*i *_= 2, 3, .. 20 AAs divided by the number of all segments in a protein (i.e., the sum of all coil, helix and strand segments). (19 features)

- *SegCount_{E,H}_P{P*_*i*_} = , the number of helix or strand segments which contain at least *P*_*i*_AAs where *P*_*i *_= 2,4,..,10% of the sequence length, divided by the total number of helix and strand segments in the input protein chain. (2*5 = 10 features)

- *SegCount_C_P{P*_*i*_} = , the number of coil segments which contain at least *P*_*i *_AAs where *P*_*i *_= 2,4,..,10% of the sequence length, divided by the number of all segments. (5 features)

- *NormSegCount_{SS*_*n*_} = , the total number of *SS*_*n *_segments divided by the total number of all secondary structure segments in the input protein chain. (3 features)

- *MaxSegLength_{SS*_*n*_} = max *Len*(*SEG*: *SEG*(*SS*_*n*_)), the maximal *SS*_*n *_segment length. (3 features)

- *NormMaxSegLength_{SS*_*n*_} = , the maximal *SS*_*n *_segment length divided by the sequence length. (3 features)

- *AvgSegLength_{SS*_*n*_} = *avgLen*(*SEG*: *SEG*(*SS*_*n*_)), the average *SS*_*n *_segment length. (3 features)

- *NormAvgSegLength_{SS*_*n*_} = , the average *SS*_*n *_segment length divided by the sequence length. (3 features)

#### Features based on the collocation of helix and strand segments in the predicted secondary structure (127 features)

The four main structural classes are based on the content and relative spatial position of the secondary structures. The preferred way to represent these collocations of the secondary structures would be to use 3D protein structure. However, since our input is only the sequence, we approximate this information using features that quantify collocation of helices (H) and strands (E) in the predicted secondary structure. We use the predicted secondary structure to annotate helix, coil and strand segments and to compute relative position of these segments in the sequence. The following features are computed:

- *HH *= *count*(*HH*), the number of helix-coil-helix motifs (two helices separated by a coil) divided by the total number of the secondary structure segments in a protein. (1 feature)

- *EE *= *count*(*EE*), the number of strand-coil-strand motifs (two strands separated by a coil) divided by the total number of the secondary structure segments in a protein. (1 feature)

- *HE *= *count*(*HE*) + *count*(*EH*), the number of strand-coil-helix or helix-coil-strand motifs (strand and helix separated by a coil) divided by the total number of the secondary structure segments in a protein. (1 feature)

- *{HH,HE,EE}_L{L*_*i*_} = , the number of helix-coil-helix, helix-coil-strand/strand-coil-helix, or strand-coil-strand motifs which include at least *L*_*i *_= 2, 3, .., 20 residues in the middle coil, divided by the total number of the secondary structure segments in a protein. (3*19 = 57 features)

- *{HH,HE,EE}_P{P*_*i*_} = , the number of helix-coil-helix, helix-coil-strand/strand-coil-helix, or strand-coil-strand motifs which include at least *P*_*i *_= 2, 4, .., 10% of a sequence length residues in the middle coil, divided by the total number of the secondary structure segments in a protein. (3*5 = 15 features)

- *MaxHCH *= max(*HC*..*H*: *count*(*H*)), the maximal number of helices among all helix-coil-helix-coil...coil-helix motifs, i.e., the maximal number of helix segments separated only by coils. (1 feature)

- *MaxECE *= max(*EC*..*E*: *count*(*E*)), the maximal number of strands among all strand-coil-strand-coil...coil-strand motifs, i.e., the maximal number of strand segments separated only by coils. (1 feature)

- *AvgHCH *= , the average number of helices in all helix-coil-helix-coil...coil-helix motifs, divided by the total number of the secondary structure segments in a protein. (1 feature)

- *AvgECE *= , average number of strands in all strand-coil-strand-coil...coil-strand motifs, divided by the total number of the secondary structure segments in a protein. (1 feature)

- *HCH_L{L*_*i*_} = , the number of helix-coil-helix-coil...coil-helix motifs with more than *L*_*i *_= 1, 2, .., 20 helices, divided by the total number of the secondary structure segments (19 features)

- *HCH_P{P*_*i*_} = , the number of helix-coil-helix-coil...coil-helix motifs with more than *P*_*i *_= 2, 4, .., 10% of all helices in a protein, divided by the total number of the secondary structure segments (5 features)

- *ECE_L{L*_*i*_} = , the number of strand-coil-strand-coil...coil-strand motifs with more than *L*_*i *_= 1, 2, .., 20 strands, divided by the total number of the secondary structure segments (19 features)

- *ECE_P{P*_*i*_} = , the number of strand-coil-strand-coil...coil-strand motifs which more than *P*_*i *_= 2, 4, .., 10% of all strands in a protein, divided by the total number of the secondary structure segments (5 features)

### Feature and classifiers selection

Feature selection was performed to select the best subset of the considered features for each structural class. This is motivated by the fact that while the considered features are generic, the individual structural classes are likely characterized by a small and specific set of descriptors. In other words, while the features describe the sequence, conservation of residues and predicted secondary structure for every protein in the same way, the structural classes can be described by a subset of these features, i.e., for a specific class some features could be irrelevant and should be discarded to improve the efficiency of the prediction model. We considered a comprehensive set of eight feature selection methods which include four methods that select feature sets and four methods that perform feature ranking. The first group includes consistency subset selection [107], wrapper-based feature selection with Naïve Bayes and SVM classifiers [108], and Correlation-based Feature Subset selection [109] (CFS) methods. The latter group includes a filter-based ReliefF algorithm [110], and three methods that perform ranking based on Symmetrical Uncertainty [111], Chi-Squared (the chi-squared statistic with respect to the class) and Gain Ratio (measure based on entropy with respect to the class) criterions. The feature selection was performed based on tenfold cross validation on the ASTRAL_training _dataset. In the case of the methods that select feature sets, individual features were ranked based on the number of folds in which they were selected. For the ranking methods the feature were ranked based on as the average rank over the ten folds.

We considered four classifiers which are based on complementary model types: nonlinear kernel-based SVM [112], probabilistic Naïve Bayes [113], linear Logistic regression [114], and instance-based *k*-Nearest Neighbor [115] (*k*-NN) with *k *= 3. The selection was also motivated by their prior successful applications in the context of the structural class predictions, i.e., Naïve Bayes based classifier was used in [81], logistic regression in [30,58,72,73], nearest neighbor in [52,67-69], and SVM in [45,47,48,50,53,54,61,62,66,72,74,75,79,82].

The quality of the prediction was reported using several measures including overall accuracy (the number of correct predictions divided by the total number of test sequences), accuracy for each structural class (number of correct predictions for a given class divided by the number of sequences from this class), Matthews's correlation coefficient (MCC) for each structural class, and generalized squared correlation (GC^2^). The MCC values range between -1 and 1, where 0 represents random correlation, and bigger positive (negative) values indicate better (lower) prediction quality for a given class. Since MCC works only for binary classification, we also reported GC^2^, which is based on χ2 statistics. The GC^2 ^values range between 0 and 1, where 0 corresponds to the worst classification (all predictions are incorrect) and 1 corresponds to the perfect classification. MCC and GC^2 ^are described in detail in [116]. During the design we selected a classifier/feature subset combination that provides the best MCC value for a given class. We used MCC since this measure, in contrast to accuracy, takes into account the unbalanced nature of the datasets, i.e., while high accuracy could be obtained for a default classification in which small class is ignored (only large class is predicted), positive MCC values assure that both small and large classes are correctly predicted.

For each structural class and each of the four considered classifiers we used the output of each of the eight feature selection methods to find the best subset of features, i.e., subset of features that provides the highest MCC value for a given classifier. For the four selection methods that generate subsets of features, we considered different subsets based on the number of folds in which a given feature was selected. In other words, for each of the four methods we generated subsets of features that were included in at least 1 cross validation fold, at least 2 folds, ...., and at least 10 folds (total of 4 × 10 = 40 feature sets). In the case of the four feature ranking methods, we started with the highest ranked features and kept adding subsequent features until the MCC values for a given classifier was increasing (total of 4 feature sets). Finally, for each of the 44 feature sets we compared results of the tenfold cross validation test on the ASTRAL_training _dataset using each of the classifiers to select the setup with the highest MCC for a given structural class.

We note that although Naïve Bayes, logistic regression and *k*-NN do not require parameterization, SVM is sensitive to parameterization. We used SVM with linear kernel and cost parameter *C *set to 1 to find the best feature set for each structural class (this default setup allows for fast generation of the model), and later we used two different kernels, polynomial and RBF, and different values of *C *to parameterize the SVM for the selected feature sets. We performed a grid search (considering values of *C *and γ for the RBF kernel, and values of *C *and exponent for the polynomial kernel) and selected the configurations that provide the highest MCC values for the tenfold cross validation on the ASTRAL_training _dataset.

Our resulting design shows that the best results for all seven classes were obtained with the SVM classifier. This is consistent with the successful prior use of this classifier for the prediction of the four major structural classes [45,47,48,50,53,54,61,62,66,72,74,75,79,82]. Table 2 summarizes the selected classifiers, i.e., it lists the results of the parameterization of the SVM classifier, and the feature selection methods together with the number of the selected features for each of the seven considered structural classes. We observe that usage of a variety of feature selection methods was proven beneficial since five out of eight of them were used to derive the final feature sets.

**Table 2:** Results of the feature and classifier selection for the considered seven structural classes.

Class	Kernel	C	Feature selection method	# of selected features
all-α	RBF (γ = 0.05)	10	Wrapper with SVM	117

all-β	RBF (γ = 0.1)	7	Wrapper with NB	53

α/β	Polynomial (exp = 2)	2	ReliefF	46

α+β	RBF (γ = 0.15)	4	CFS	163

Multi-domain	Polynomial (exp = 1.5)	0.5	Wrapper with SVM	105

Membrane	Polynomial (exp = 1.5)	10	Symmetrical Uncertainty	46

Small	Polynomial (exp = 2.5)	15	Wrapper with NB	18

### Classification

Once the user selects the classes that (s)he would like to consider, the input sequence is converted into the feature space and the corresponding feature sets are passed to the classifiers for each of the selected classes. Each of the classifiers returns a probability that the input sequence belongs to a given class. The prediction corresponds to the class that is associated with the highest probability. This type of aggregation allows the user to select any combination of the classes.

## Results and Discussion

This section includes discussion of the selected feature sets, reports results of the proposed MODAS method on the independent test set ASTRAL_test _and compares them with results provided by several competing solutions, and compares the results of the proposed and over two dozens of existing methods for the prediction of the structural classes on five benchmarking datasets including D2230, 25PDB, D1189, D675, and D498. We emphasize that all considered datasets, except D498, are characterized the twilight zone pairwise sequence similarity (which is also true for the pair of the ASTRAL_test _and ASTRAL_training _datasets). We report the overall accuracy, accuracies and MCC values for each structural class, and the GC2 values.

### Discussion of the selected features

The selected features are summarized using Tables 3 and 4. The former table shows the number of selected features for each of the five feature set and for each structural class. The latter table presents details related to features computed from the predicted secondary structure focusing on different types of the secondary structures.

**Table 3:** Number of features selected for each structural class for different categories of features.

Class	AA sequence	PSSM	PSSM and predicted secondary structure	Predicted secondary structure	Collocations of H and E segments	Total
α	8	26	52	21	10	117

β	2	28	9	8	6	53

α/β	0	0	17	17	12	46

α+β	2	11	101	27	22	163

Multi-domain	3	17	43	26	16	105

Membrane	6	16	18	6	0	46

Small	6	6	3	3	0	18

**Table 4:** Number of the selected features for the features computed from the predicted secondary structure.

Class	AA+PSSM	Secondary structure (including PSSM)			Collocations of helical and strand segments		
		**C**	**H**	**E**	**Helices (HH, HCH)**	**Strands (EE, ECE)**	**Helices and Strands (HE, HCE)**

α	34	22	17	34	8	1	1

β	30	12	3	2	2	2	2

α/β	0	1	20	13	2	1	9

α+β	13	5	50	73	18	4	0

Multi-domain	20	17	24	28	8	5	3

Membrane	22	0	24	0	0	0	0

Small	12	2	2	2	0	0	0

We observe that only a few sequence based features are used by the proposed MODAS method. More specifically, although the total number of features in this set includes 39 only between 0 and 8 of them are used by the seven classifiers. The most frequently used source of information is the PSSM in combination with the predicted secondary structure. For almost all classes, including all-α, all-β, α+β, multi-domain, membrane and small proteins, over half of the features are computed using PSSM. This confirms that the conservation of the residues provides higher quality information than their presence. In the case of the remaining α/β class the majority of features are based on the predicted secondary structure. We also note that a few other classes, such as all-α, α+β, multi-domain and membrane proteins, heavily utilize the information concerning the predicted secondary structure in connection with the PSSM. The popularity of the features derived from the secondary structure stems from the fact that the structural classes are de facto defined based on the secondary structures.

The predictor for the all-α class uses large number of features from PSSM and PSSM combined with the predicted secondary structure. This shows that residue conservation is an important factor that distinguishes between all-α and other classes. We also observe that these features utilize information about both helix and strand segments, where the strand segments are likely used to indicate non all-α proteins. Finally, this feature set includes 8 features based on the helix-coil-helix motifs that occur in virtually all proteins from this class.

Most of the features for the all-β class are again based on the PSSM. This feature set also includes features that quantify the amount of helix-coil-helix (likely to exclude non all-β classes) and strand-coil-strand segments (which are specific to the proteins from the all-β class) and a relatively large number of coil-based features. The latter is likely due to the fact that proteins from the all-β class include relatively large number of β-sheets which incorporate larger number of coils (when compared with other classes) that connect individual strand segments that make up the β-sheet.

The α/β class incorporates a relatively large number of features that quantify the occurrence of the helix-coil-strand and strand-coil-helix motifs. This agrees with the definition of this class that incorporates structures in which helices and strands are interspersed. Such spatially scattered secondary structures are likely to also alternate in the sequence.

The largest number of features was selected for the α+β class. This is likely because this class is the hardest to predict among the four major classes, e.g., 17 out of 18 structural class prediction methods that were recently compared in [79] provide the lowest prediction quality for this class when compared with the predictions for the all-α, all-β and α/β classes. Most of the features utilized by the α+β classifier are based on the PSMM combined with the predicted secondary structure. All of the features that exploit collocation of the helix and strand segments are based on either collocation of helix (helix-coil-helix) or strand (strand-coil-strand) segments. This is motivated by the definition of this class that includes protein in which secondary structures are segregated.

The multi domain proteins have structures that combine characteristics of the four major structural classes since different domains may fold into structures characteristic to different classes. This is likely the reason why this class uses relatively equal number of features coming from different sources, like the PSSM and the predicted secondary structure, and why the secondary structure based features equally cover all three structure types (coils, strands and helices).

The membrane proteins include long transmembrane α-helices and this is the likely the reason why the corresponding classifier makes use of 24 out of 46 features that are based on the predicted helices. As in the case of most of the other classes, features used to classify membrane proteins also heavily rely on the residue conservation.

We note that although the small protein class includes short protein chain, the feature that measures the sequence length was not selected for the corresponding classifier. This is likely since several other classes also include short chains, but their secondary structure fits the definition of a given class rather than being composed mostly of coils which is characteristic for the small proteins class. The features for this class come from different sources including the sequence, the PSSM and the predicted secondary structure. We observe that helix/strand collocation based features were not selected for this class; again, this is likely since these proteins are mostly composed of coils.

**Figure 4:**
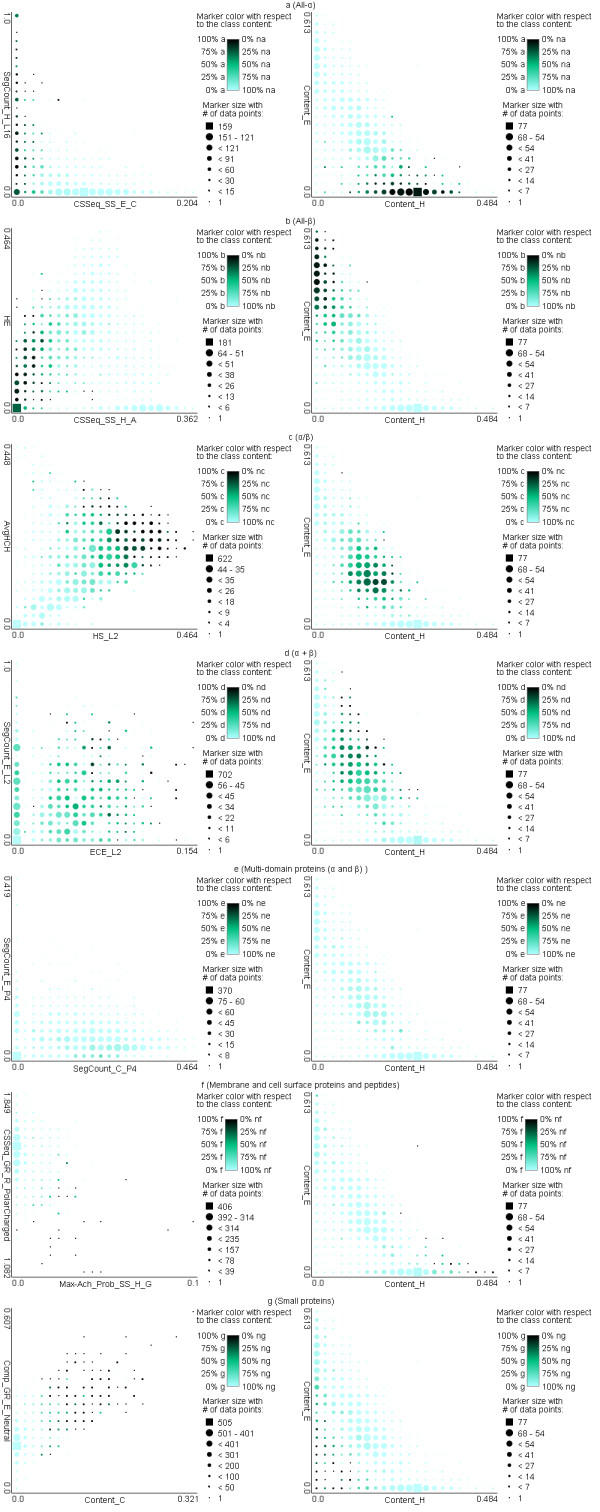
**Scatter plots for two representative features for each structural class (left column) and helix and strand contents (right column) for a) all-α; b) all-β; c) α/β; d) α+β; e) multi-domain; f) membrane and cell surface proteins; and g) small proteins classes**. The plots were computed on the ASTRAL_training_dataset and they use markers with colors and shapes that indicate the class and number of protein chains for a given combination of the values of the two features, respectively. The larger the marker is the more chains are found for the corresponding values of the two features. The darker the shading of the marker is the larger the fraction of the chains that correspond to the target class is for the given values of the two features.

We also discuss the most useful features for prediction of each of the considered seven structural classes. We select two representative features for each class and use a scatter plot of their values to explain their relation with the classes. The selection of the features is based on their correlation with the classes (which should be high) and correlation with each other (which should be relatively low to limit their overlap). The first feature was selected based on the largest values of its biserial correlation with the class labels (a given class vs. the remaining classes). The remaining features were ranked based on their biserial correlation coefficients and the top ranked feature for which the Pearson correlation coefficient with the first feature is smaller than 0.7 was selected as the second feature. We also compare the scatter plots for these two features with the scatter plots when using helix and strand content to discriminate between the classes. This is motivated by the fact that some older structural class assignment methods performed the class assignment using the secondary structure content rather than the spatial arrangement of the secondary structures which comes from the tertiary structure [43]. Figure 4 presents the corresponding 14 scatter plots.

The two representative features for the all-α class are CSSeq_SS_E_C (normalized conservation scores for the substitution into Cys for AAs that were predicted as strands) and SegCount_H_L16 (normalized count of long helical segments in the predicted secondary structure). We observe that proteins with high SegCount_H_L16 values and proteins with low values of CSSeq_SS_E_C likely belong to the all-α class. This is supported by the fact that all-α proteins are characterized by significant helix content and thus they include relatively large number of long helices. The CSSeq_SS_E_C feature shows that all-α proteins include virtually no strands in which Cys is conserved. Costantini and colleagues have observed that Cys has strong propensity to form strands and is more prevalent among the proteins from all-β class [117] and thus proteins that include strands with conserved Cys are unlikely to belong to all-α class. The right-hand-side plot in Figure 4a shows that the all-α proteins are characterized, as expected, by a high content of helices and a low content of strands. At the same time, we note that some non all-α proteins (right lower corner of the scatter plot) could be misclassified using this criteria, which shows that the two representative features used in the proposed method likely provide better discriminatory power.

The two features selected for the all-β class (Figure 4b) include HE (the number of strand-coil-helix or helix-coil-strand motifs in the predicted secondary structure) and CSSeq_SS_H_A (normalized conservation scores for the substitution into Ala for AAs that were predicted as helices). The proteins from this class have low CSSeq_SS_H_A and medium to low HE values for chains for which CSSeq_SS_H_A values are close to zero. The HE feature is motivated by the fact that all-β proteins include relatively large number of strands and a low number of helices and thus strand-coil-helix or helix-coil-strand motifs are less likely to occur in these proteins. The CSSeq_SS_H_A feature shows that the all-β class includes chains that have very few helices with conserved Ala. This is supported by the work in [117] which shows that Ala has strong propensity to form helices and occurs relatively more frequently in proteins from the all-α class, which suggests that chains that include helices with conserved Ala are unlikely to belong to the all-β class.

The proteins from the α/β class are characterized by average values of AvgHCH (the average number of helices in all helix-coil-helix-coil...coil-helix motifs in the predicted secondary structure) and high values of HE_L2 (the number of helix-coil-strand or strand-coil-helix motifs which includes at least 2 residues in the middle coil) features. The HE_L2 indicates that the proteins from this class have the helices and strands interspersed in the sequence and AvgHCH shows that they do not include secondary structures with no consecutive helices and with many consecutive helices. The latter shows that α/β class includes proteins with helices, but they are less likely to form long helix-coil-helix-coil...coil-helix motifs.

The two representative features for the α+β class include SegCount_E_L2 (the number of strand segments which contain at least 2 AAs) and ECE_L2 (the number of strand-coil-strand-coil...coil-strand motifs with more than 2 strands). Protein from this class have average to high values of both features which is motivated by the observation that they have strands (SegCount_E_L2 features excludes beta bridges and includes extended strands that likely form sheets) and the strands and helices are segregated, i.e., that strands co-occur closely in the sequence, which results in high values of ECE_L2. We observe that usage of the content leads to a significant overlap between the proteins from the α/β and α+β classes, see the right-hand-side plots in Figures 4c and 4d. At the same time, the proposed method uses different features for different classes, which can potentially provide better discrimination between these two classes when compared to using the content. The representative features for the α/β and α+β classes quantify the spatial relation of the helices and strands (which is done based on their co-occurrence close by in the sequence) which, in our opinion, better captures the characteristics of these two classes when compared with the content.

The scatter plot for the multi domain proteins class shows no clear trends since the number of proteins in this class is small, only 53 out of 3132 in the ASTRAL_training _dataset, and since the best feature for this class has relatively small biserial correlation value of 0.12. This is likely due to the significant overlap between this class and other classes, i.e., individual domains in these proteins belong to different structural classes. We observe that proteins from this class have relatively high value of SegCount_C_P4 (the number of coil segments which length is at least 4% of the sequence length) combined with low value of SegCount_E_P4 (the number of strand segments which length is at least 4% of the sequence length). This suggests that on average they include longer coil segments and a few or none longer strands when compared with other classes. We note that similar structures occur also for chains from other classes, i.e., the markers in Figure 4e have only relatively light shading. We also observe that the usage of helix and strand contents results in the scatter plot with even lighter shading of the markers.

The membrane and cell surface proteins are best described using CSSeq_GR_R_PolarCharged (sum of the normalized conservation scores for the substitution into polar charged residues that include Asp, Glu, His, Lys, and Arg) and Max-Ach_Prob_SS_H_G (the difference between the maximal and the achieved probability of the occurrence of Gly residues predicted as helices) features (Figure 4f). These proteins are characterized by high values of Max-Ach_Prob_SS_H_G, which is motivated by the inclusion of transmembrane helices [118] and by frequent presence of Gly in membrane proteins [119]. This class is also associated with medium to low values of CSSeq_GR_R_PolarCharged, which is supported by prior research that shows that Asp, Arg, Lys, Gln, Asn, Glu, Pro, Ser, Thr, Gly, and His are characterized by low (in descending order) propensity to form membrane regions based on the membrane propensity scale from [120]; in other words, the existence of the conserved residues of the above type suggests that the corresponding chain is less likely to be associated with the membrane regions in the protein chain.

Lastly, the high values of Comp_C (content of the predicted coils) together with above average values of Comp_GR_E_Neutral (composition of the neutral residues that include Cys, Gly, His, Ser, and Trp) features are shown to be associated with the small proteins class. The former agrees with the strand and helix content scatter plot (see right-hand-side plot in Figure 4g) that shows that small proteins usually include only a few helix and strand structures. According to Costantini and coworkers Gly, His, and Ser are shown to be among the amino acids with high propensity to form coils [117], which is a likely reason why Comp_GR_E_Neutral feature was selected.

### Results for the independent test set ASTRAL_test_

The proposed prediction system was trained using the ASTRAL_training _dataset and tested using the ASTRAL_test _database. A summary presented in Table 5 shows results for three configurations of the proposed MODAS method that include prediction of the four major classes, six classes that exclude the small proteins class, and prediction of all seven classes. For each setup we use only the instances from the selected classes to perform the test.

**Table 5:** Experimental results for the test on the independent ASTRAL_training_dataset for the proposed MODAS method that considers the 4 major structural classes, 6 classes excluding the small proteins class, and all 7 considered classes.

# of classes	Accuracy								MCC							**GC**^2^
	**α**	**β**	**α/β**	**α+β**	**multi-domain**	**membrane and cell surface**	**smal**l	**Overall**	**α**	**β**	**α/β**	**α+β**	**multi-domain**	**membrane and cell surface**	**smal**l	

4	94.06	83.38	85.01	71.47				83.01	0.92	0.79	0.78	0.61				0.63

6	93.28	82.63	82.20	71.07	26.42	57.97		80.24	0.90	0.78	0.75	0.61	0.22	0.74		0.49

7	91.72	82.18	82.20	70.29	26.42	57.97	84.26	79.89	0.89	0.78	0.76	0.60	0.22	0.75	0.84	0.52

The results show that the accuracy is around 83% for the prediction of the four major classes and close to 80% when considering the 7 classes. This moderate drop in accuracy is attributed to the predictions for the multi-domain proteins class which obtains the lowest accuracies. We note that positive MCC values indicate that the proposed model provides predictions that are always better than random. Most importantly, in spite of the twilight zone similarity between training and testing sequences we observe that the proposed method is characterized by good performance for all classes except the multi-domain proteins class, which is supported by the MCC and GC^2 ^values of above 0.6 and 0.5, respectively. The all-α class is the easiest to predict. The corresponding predictions for all three configurations are characterized by accuracy of above 91% and MCC of 0.89 or higher. The predictions for the α/β and all-β classes have similar quality with accuracies ranging between 82 and 85% and MCC between 0.75 and 0.79. The predictions of the small proteins class are also characterized by a relatively high accuracy and MCC. We observe that inclusion of this class, see the results for the 6 and 7 classes in Table 5, results in a slight drop in the quality of the prediction of the all-α, all-β, and α+β classes. This suggests existence of an overlap between these classes and the small proteins class. The relatively poor scores for the multi-domain proteins class are likely due to the small size of this class and since proteins from this class consist of domains that likely belong to different structural classes. Although the accuracy of the prediction of the membrane proteins is at 58%, we emphasize that relatively high MCC value of 0.75 indicates that the proposed method performs well for this class. The results for this class should be considered successful given that this class is significantly underrepresented in the datasets, i.e., membrane proteins account for only 2.2% of proteins in both the ASTRAL_training _and the ASTRAL_test _databases.

We also compare the results obtained by the proposed MODAS method on the ASTRA_*test *_dataset with the results of two recent representative methods that were designed to work with low identity sequences, SCPred [79] and SCEC [75]. Both of these methods use SVM to perform predictions and they are shown to provide favorable prediction quality with compared with other existing structural class predictors (see results in the "Comparison with the existing structural class predictors" section). SCPred is the only existing method that uses predicted secondary structure to predict the structural classes and SCEC uses PSMM to compute the predictions. These two methods predict only the four major classes and thus we compare the performance considering only these classes. We removed sequences from the three minor classes and sequences with less than 30 residues from the training and test sets since SCEC cannot provide predictions for such short chains. The SCPred algorithm was trained both on the original 25PDB dataset as it was done by the authors of this method [79], and we also retrained this method using ASTRAL_training _dataset. In the case of the SCEC algorithm we used the corresponding web server to perform predictions. We assumed that the user of the MODAS system may not know how many classes should be considered in the test and thus we included the results when prediction was made for only the 4 major classes, the 6 classes (excluding the small proteins), and all 7 classes. The results are presented in Table 6.

**Table 6:** Results of the experimental comparison of the proposed MODAS method and the competing SCEC and SCPRED methods on the ASTRAL_test_dataset with the four major structural classes.

Algorithm	Training dataset	Accuracy					MCC				**GC**^2^
		**α**	**β**	**α/β**	**α+β**	**Overall**	**α**	**β**	**α/β**	**α+β**	

MODAS with 4 classes	ASTRAL_training_	**94.05**	**83.48**	**85.12**	**71.47**	**83.05**	**0.92**	**0.79**	**0.78**	0.61	0.63

MODAS with 6 classes	ASTRAL_training_	93.27	82.73	82.31	71.07	81.84	**0.92**	**0.79**	0.77	**0.62**	**0.64**

MODAS with 7 classes	ASTRAL_training_	91.71	82.27	82.31	70.29	81.17	0.91	0.79	0.77	0.61	**0.64**

SCPRED	ASTRAL_training_	93.13	78.33	83.38	64.27	79.14	**0.92**	0.77	0.70	0.52	0.57

SCPRED	25PDB	92.81	79.09	80.05	63.74	78.36	**0.92**	0.78	0.67	0.51	0.56

SCEC	Web server	75.74	72.73	78.42	28.14	62.80	0.65	0.55	0.59	0.22	0.29

The MODAS method was used to make predictions for all 7, 6 (excluding the small proteins class), and the 4 major classes. The SCEC method was trained on the ASTRAL_training _with the proteins from the 4 major classes (this method can handle only prediction of the 4 classes) and on the 25PDB dataset based on results in [53]. The SCEC predictions were generated using the web server at http://biomine.ece.ualberta.ca/Structural_Class/SCEC.html. Bold font indicates the best results.

The MODAS method is shown to provide favorable quality for the prediction of the 4 classes. The quality of the results generated by the proposed method is slightly lower when using predictions that consider more classes, but the overall accuracy and GC^2 ^are still higher than the quality provided by both competing solutions even when using the model that predicts all 7 classes. The accuracy improvements of the best MODAS model that predicts 4 classes over the best results from other methods equal 0.9%, 4.4%, 1.7%, and 7.2% for the all-α, all-β, α/β, and α+β classes, respectively. This translates into 0.9/(100-93.1) = 0.9/6.9 = 13%, 4.4/20.9 = 21%, 1.7/16.6 = 10%, and 7.2/35.7 = 20% error rate reductions, respectively, when compared with the error produced by the best performing competing method. The corresponding error rate reduction for the overall accuracy equals 3.9/(100-79.1) = 19%. The most encouraging improvements that are measured using MCC concern α/β and α+β classes where the MODAS method is better by at least 0.08 when compared with the best existing method. We also observe that SCPred performs slightly better when trained on the bigger ASTRAL_training _dataset. The SCEC provides the lowest ranked predictions among the considered methods.

### Comparison with the existing structural class predictors

The side-to-side comparison with recently proposed structural class prediction methods is based on the tests on three popular benchmarking datasets, 25PDB, D1189 and D675, which are characterized by the low sequence identity. These sets were used to test methods that predict the 4 major classes and thus the proposed MODAS method is also setup to predict these 4 classes. We also use the D2230 dataset to compare with the most recent structural class predictor that considers the 7 classes [83]. Following the prior works in this area we use jackknife test to measure the performance. The selection of this test strategy is motivated by the work in [23,121] which shows that jackknife is deemed the most objective as it always yields a unique result for a given dataset and that this test is increasingly used to examine the accuracy of various predictors. In this test all but one sequence are used to train the proposed classification system (using parameters and features identical to those discusses in the Materials and Methods section) and the remaining sequence is used to perform the test; this process is repeated to use each sequence from the dataset once as the test sequence.

Table 7 that concerns tests on the 25PDB dataset shows that the proposed MODAS method outperforms all other methods. There are only two methods that provide the overall accuracy of over 65%, which include different variants of the SCPRED method [79] and MODAS, and both of them use SVM classifiers and predicted secondary structure. This suggests that the predicted secondary structure provides a useful source of information and that SVM classifiers provide favorable prediction quality for this prediction task. Comparison with the SCPRED reveals that the proposed method obtains higher overall accuracy and higher accuracy for the all-β and α/β classes. The error rate reduction obtained by MODAS when compared with the second best SCPRED on this dataset equals 1.7/(100-79.7) = 8%. We note that the proposed predictor was designed to maximize the MCC values (the feature selection and classifier parameterization were performed to maximize the MCC values) and as a result it provides the best predictions for the 25PDB dataset according to this quality index. The biggest improvement, when compared with SCPRED, was obtained for the α/β class which is likely due to the introduction of novel features that describe collocation of helix and strand segments in the predicted secondary structure.

**Table 7:** Results of the experimental comparison between the proposed MODAS method and competing structural class prediction methods on the 25PDB dataset.

Classifier used (name of the method, if any)	Feature vector (# features)	Reference	Accuracy					MCC				**GC**^2^
			**α**	**β**	**α/β**	**α+β**	**overal**l	**α**	**β**	**α/β**	**α+β**	

SVM with 1st order polyn. kernel	autocorrelation (30)	73	50.1	49.4	28.8	29.5	34.2	0.16	0.16	0.05	0.05	0.02

Multinomial logistic regression	custom dipeptides (16)	58	56.2	44.5	41.3	18.8	40.2	0.23	0.20	0.31	0.06	0.05

Bagging with random tree	CV (20)	54	58.7	47.0	35.5	24.7	41.8	0.33	0.26	0.22	0.06	0.06

Information discrepancy	tripeptides (8000)	59, 60	45.8	48.5	51.7	32.5	44.7	0.39	0.39	0.25	0.06	0.11

LogicBoost with decision tree	CV (20)	46	56.9	51.5	45.4	30.2	46.0	0.41	0.32	0.32	0.06	0.10

Information discrepancy	dipeptides (400)	59, 60	59.6	54.2	47.1	23.5	47.0	0.46	0.40	0.24	0.04	0.12

LogitBoost with decision stump	CV (20)	54	62.8	52.6	50.0	32.4	49.4	0.49	0.35	0.34	0.11	0.13

SVM with 3rd order polyn. kernel	CV (20)	54	61.2	53.5	57.2	27.7	49.5	0.46	0.35	0.39	0.11	0.13

SVM with Gaussian kernel	CV (20)	47	68.6	59.6	59.8	28.6	53.9	0.52	0.42	0.43	0.15	0.17

Multinomial logistic regression	custom (66)	73	69.1	61.6	60.1	38.3	57.1	0.56	0.44	0.48	0.21	0.21

Nearest neighbor	Composition of tripeptides (8000)	52	60.6	60.7	67.9	44.3	58.6	---	---	---	---	---

SVM with RBF kernel	custom (34)	72	69.7	62.1	67.1	39.3	59.5	0.60	0.50	0.53	0.21	0.25

Multinomial logistic regression	custom (34)	72	71.1	65.3	66.5	37.3	60.0	0.61	0.51	0.51	0.22	0.25

StackingC ensemble	custom (34)	72	74.6	67.9	70.2	32.4	61.3	0.62	0.53	0.55	0.22	0.26

Linear logistic regression	custom (58)	30	75.2	67.5	62.1	44.0	62.2	0.63	0.54	0.54	0.27	0.27

SVM with 1st order polyn. kernel	custom (58)	30	77.4	66.4	61.3	45.4	62.7	0.65	0.54	0.55	0.27	0.28

SVM with RBF kernel	custom (56)	61	76.5	67.3	66.8	45.8	64.0	0.62	0.51	0.50	0.28	---

Discriminant analysis	custom (16)	78	64.3	65.0	61.7	65.0	64.0	---	---	---	---	---

SVM with Gaussian kernel	custom (8 PSI Pred based)	79	**92.6**	80.6	73.4	68.5	79.1	0.87	**0.79**	0.67	0.54	0.54

SVM with Gaussian kernel	PSI Pred based (13)	79	**92.6**	79.8	74.9	69.0	79.3	0.87	**0.79**	0.68	0.55	0.55

SVM with RBF kernel (SCPRED)	custom (9)	79	**92.6**	80.1	74.0	**71.0**	79.7	0.87	**0.79**	0.69	0.57	0.55

SVM with polynomial or RBF kernels (MODAS)	custom(117, 53, 46, 163)	this paper	92.3	**83.7**	**81.2**	68.3	**81.4**	**0.88**	**0.79**	**0.76**	**0.58**	**0.58**

The results were obtained using jackknife test. The methods are ordered by their average accuracies which coincide with the GC^2 ^scores. Best results are shown in bold and "---" indicates results that were not reported by the original authors and which cannot be duplicated.

Results shown in Tables 8 and 9, which concern jackknife tests on the D1189 and D675 datasets, respectively, are consistent with the results on the 25PDB dataset. The MODAS method outperforms all competing methods as measured by the overall accuracy. The only method that provides similar prediction quality is again SCPRED. Results show that accuracy provided by MODAS is better than the accuracy of SCPRED by 2.9% and 0.5% on the D1189 and D675 datasets, respectively. The proposed method provides substantial improvements over SCPRED for the prediction of the α+β class. The SCEC predictor, which utilizes PSSM generated with PSI Blast as its input, provides the third best results on both of these datasets. This demonstrates that evolutionary information provides a better source of information for the prediction of the structural class when compared with the sequence of the input protein that is used as an input by all lower ranked methods. We note that the size of the dataset used to build PSSM would likely impacts the prediction quality, as it was demonstrated for the secondary structure predictions [106]. Larger size of the dataset may induce better prediction performance, which could explain a portion of the improvements of the MODAS method that was trained using relatively recent version of the nr database, when compared with other predictors, including SCEC and SCPRED, which used smaller datasets. We could not provide MCC and GC^2 ^values for results on these two datasets (as well as for the D498 dataset) since they were not provided by the authors of the existing methods.

**Table 8:** Results of the experimental comparison between the proposed MODAS method and competing structural class prediction methods on the D1189 dataset.

Classifier used (name of the method, if any)	Feature vector	Reference	Accuracy				
			α	β	α/β	α+β	overall
SVM	AA composition, autocorrelations, and physicochemical properties	73	-	-	-	-	52.1

Bayesian classifier	AA composition	81	54.8	57.1	75.2	22.2	53.8

Logistic regression	AA composition, autocorrelations, and physicochemical properties	73	60.2	60.5	55.2	33.2	53.9

SVM	AA and polypeptide composition, physicochemical properties	45	-	-	-	-	54.7

Nearest neighbor	Pseudo-amino acid composition	67	48.9	59.5	81.7	26.6	56.9

Ensemble	AA composition, autocorrelations, and physicochemical properties	72	-	-	-	-	58.9

Nearest neighbor	Composition of tripeptides	52	-	-	-	-	59.9

IB1	PSI Blast based collocated AA pairs	75	65.3	67.7	79.9	40.7	64.7

Discriminant analysis	custom	78	62.3	67.7	63.1	66.5	65.2

SVM with RBF kernel (SCEC)	PSI Blast based collocated AA pairs	75	75.8	75.2	82.6	31.8	67.6

SVM with RBF kernel (SCPRED)	custom	79	89.1	86.7	**89.6**	53.8	80.6

SVM with polynomial or RBF kernels (MODAS)	custom	this paper	**92.3**	**87.1**	87.9	**65.4**	**83.5**

The results were obtained using jackknife test. The methods are ordered by their average accuracies. Best results are shown in bold and "---" indicates results that were not reported by the original authors and which cannot be duplicated.

**Table 9:** Results of the experimental comparison between the proposed MODAS method and competing structural class prediction methods on the D675 dataset.

Classifier used (name of the method, if any)	Feature vector	Reference	Accuracy				
			α	β	α/β	α+β	overall
Bayesian classifier	AA composition	81	53.5	42.3	68.3	28.3	48.0

IB1	PSI Blast based collocated AA pairs	75	54.9	47.4	68.9	35.0	51.5

SVM with RBF kernel (SCEC)	PSI Blast based collocated AA pairs	75	74.3	59.6	79.7	34.5	61.5

SVM with RBF kernel (SCPRED)	custom	79	89.1	**81.8**	**90.4**	58.2	79.5

SVM with polynomial or RBF kernels (MODAS)	custom	this paper	**89.9**	**81.8**	84.2	**65.9**	**80.0**

The results were obtained using jackknife test. The methods are ordered by their average accuracies. Best results are shown in bold.

We also compare MODAS with methods that were tested on datasets with unspecified sequence identity between the test and the training sequences. The results of the jackknife test on the D498 dataset are presented in Table 10. The proposed method again achieves the highest accuracy (96.8%) among all competing methods that were tested on this dataset. We observe that the lowest accuracy for this dataset is around 89%. The accuracy of 94.9% obtained by the third best SCEC method demonstrates that it is easier to obtain high predictive performance on this protein set when compared with the datasets with lower sequence identity, i.e., SCEC achieves 63-67% accuracy for the low-similarity datasets. Based on the observations from a recent study by Kurgan and Homaeian [73], the high levels of accuracy are most likely due to relatively high pairwise sequence similarity of the D498 dataset, see Datasets section. On the other hand, the differences between the accuracy on the low and the high-similarity datasets for the SCPRED and MODAS methods are smaller than for the SCEC. This is most likely since these methods were designed using low sequence identity datasets.

**Table 10:** Results of the experimental comparison between the proposed MODAS method and competing structural class prediction methods on the D498 dataset.

Classifier used (name of the method, if any)	Feature vector	Reference	Accuracy				
			α	β	α/β	α+β	Avg
Component-coupling	AA composition	70	93.5	88.9	90.4	84.5	89.2

Neural network	AA composition	80	86.0	96.0	88.2	86.0	89.2

Rough sets	AA composition and physicochemical properties	49	87.9	91.3	**97.1**	86.0	90.8

SVM with RBF kernel (SCPRED)	custom	79	94.9	91.7	94.2	86.1	91.5

SVM	AA composition	82	88.8	95.2	96.3	91.5	93.2

Fuzzy k-nearest neighbor algorithm	protein sequence	68	95.3	93.7	97.8	88.3	93.8

Nearest Neighbor (NN-CDM)	protein sequence	69	96.3	93.7	95.6	89.9	93.8

LogitBoost	AA composition	71	92.5	96.0	97.1	93.0	94.8

SVM with RBF kernel (SCEC)	PSI-BLAST based p-collocated AA pairs	75	**98.0**	93.3	95.6	93.4	94.9

IB1	PSI-BLAST based p-collocated AA pairs	75	95.0	95.8	97.8	94.2	95.7

SVM with polynomial or RBF kernels (MODAS)	custom	this paper	96.7	**97.5**	95.6	**97.1**	**96.8**

The results were obtained using jackknife test. The methods are ordered by their average accuracies. Best results are shown in bold.

Table 11 compares the proposed method with the PseAA method [83] on the D2230 dataset when considering classification into the 7 classes. Although the authors of PseAA provided only the overall accuracy of their method on this dataset, we present all quality index values obtained by the proposed MODAS method. The overall accuracy of predictions generated by MODAS is better by 22.6% when compared with PseAA. This dataset includes 16.8%, 14.3%, 32%, 28.9%, 1.1%, 2.0%, and 4.9% sequences from the all-α, all-β, α/β, α+β, multi-domain, membrane and cell surface, and small protein classes, respectively. The accuracies obtained by MODAS show that our predictions are substantially better than a random chance in spite of the heavily unbalanced nature of the dataset. We note that the quality of the predictions obtained on this dataset is consistent with the results on the other benchmark datasets that are presented above.

**Table 11:** Results of the experimental comparison between the proposed MODAS and PseAA methods on the D2230 dataset.

**Method**	**Accuracy**								**MCC**							**GC**^2^
	**α**	**β**	**α/β**	**α+β**	**multi-domain**	**membrane and cell surface**	**small**	**Overall**	**α**	**β**	**α/β**	**α+β**	**multi-domain**	**membrane and cell surface**	**small**	
MODAS	90.6	78.9	85.2	70.6	33.3	45.5	85.2	80.0	0.88	0.77	0.75	0.61	0.34	0.55	0.87	0.49
PseAA	---	---	---	---	---	---	---	57.4	---	---	---	---	---	---	---	---

The results were obtained using jackknife test. "---" indicates results that were not reported by the original authors and which cannot be duplicated.

The high quality of the results provided by SCEC and SCPRED supports our choice to use the evolutionary information encoded in PSSM and the predicted secondary structure as inputs for the proposed MODAS method. The above results demonstrate that MODAS consistently, over multiple datasets, outperforms competing approaches and that it is capable of providing high quality predictions for both the 4 major classes and the 7 classes.

## Conclusions

This work addresses lack of structural class predictors that consider seven structural classes, as defined in SCOP, and which are characterized by high prediction quality when applied to problems that involve query sequences that share twilight-zone similarity with the sequences used to develop the prediction model. This is motivated by the fact that prediction for the low-similarity sequences has applications in the detection of the remote homologues.

We propose a prediction method that applies SVM classifier on a set of features that are computed from the input protein sequence. Our design incorporates novel features that utilize sequence-derived information that includes PSSM computed with PSI Blast and secondary structure predicted with PSI Pred. We performed a comprehensive feature selection and classifier selection and parameterization procedure to optimize the quality of the predictions. The proposed method is the first to provide modular design in which a separate classifier is created for each class.

An extensive empirical evaluation of the proposed MODAS method that includes tests on 5 twilight-zone and 1 high-similarity datasets and comparison with over two dozens of modern existing structural class predictors shows that MODAS achieves the best overall accuracies for predictions of both the 4 major structural classes (all-α, all-β, α/β, and α+β) and the 7 classes (the 4 classes plus multi-domain, membrane and cell surface, and small protein classes). MODAS is shown to achieve accuracy of over 80% and GC^2 ^scores of over 0.5. The main advantages of the proposed method include (1) the high quality of the predictions for problems involving low sequence similarity datasets; (2) availability of predictions for 7 structural classes (in contrast to predictions offered by the majority of the existing methods that consider only the 4 major classes); and (3) modularity which allows the user to select any subsets of the 7 classes that will be considered as the possible outcomes for the query sequence. In particular, we observe that MODAS provides accurate predictions for the membrane and cell surface proteins, which is an important class that is not considered by the majority of the existing predictors. The improved quality stems from the usage of the two important sequence-derived sources of information, the predicted secondary structure and the evolutionary information, and the development of novel features that express collocation of the secondary structure segments in the protein sequence and that combine evolutionary and secondary structure information. The results also suggest that the information extracted from the secondary structure that is predicted along the protein chain can be successfully used to predict structural classes that are defined based on the spatial arrangement of the secondary structures.

A web server that implements the MODAS method is available at http://biomine.ece.ualberta.ca/MODAS/. This server limits the number of input sequences to 10. In the case of the larger sequence sets, the interested user is asked to contact the corresponding author. The web server was trained on the 1.73 version of the ASTRAL database with less than 20% sequence similarity (i.e. merged ASTRAL_test _and ASTRAL_training _datasets).

## Abbreviations

(3D): Three dimensional; (AA): Amino acid; (CATH): Class, Architecture, Topology and Homologous superfamily; (CFS): Correlation-based Feature Subset selection; (GC2): generalized squared correlation; (k-NN): k-Nearest Neighbor; (MCC): Matthews's correlation coefficient; (MODAS): MODular Approach to Structural class prediction; (PSSM): position specific scoring matrix; (SCOP): Structural Classification of Proteins; (SVM): support vector machine.

## Authors' contributions

MJM contributed to the conception of the proposed method, designed and implemented the feature sets and the classifiers, performed the tests, implemented the web server, contributed to the evaluation and interpretation of the results, and wrote the manuscript. LK contributed to the conception of the proposed method and the design of the feature sets and the classifier, helped in performing the tests, contributed to the evaluation and interpretation of the results, and wrote the manuscript. Both authors have read and approved the final version of the manuscript.
